# Regioselective Synthesis of *Bis*(2-halo-3-pyridyl) Dichalcogenides (E = S, Se and Te): Directed Ortho-Lithiation of 2-halopyridines

**DOI:** 10.1155/2007/69263

**Published:** 2007-03-25

**Authors:** K. K. Bhasin, Neelam Singh, Shivani Doomra, Ekta Arora, Ganga Ram, Sukhjinder Singh, Yogesh Nagpal, S. K. Mehta, T. M. Klapotke

**Affiliations:** ^1^Department of Chemistry, Panjab University, Chandigarh 160 014, India; ^2^Department of Chemistry, Chaudhary Devi Lal University, 125055 Sirsa, Haryana, India; ^3^Department of Chemistry and Biochemistry, Ludwig-Maximilians University, 81377 Munich, Germany

## Abstract

A novel method for the preparation of hitherto unknown symmetrical bis(2-halo-3-pyridyl) dichalcogenides (E = S, Se and Te) by the oxidation of intermediate 2-halo-3-pyridyl chalcogenolate, prepared by lithiation of 2-halo pyridines using lithium diisopropylamine is being reported. All the newly synthesized compounds have been characterized through elemental analysis employing various spectroscopic techniques, namely, NMR (^1^H, ^13^C, ^77^Se), infrared, mass spectrometry, and X-ray crystal structures in representative cases.

## 1. INTRODUCTION

Organoselenium and organotellurium compounds are finding renewed interest as synthetic reagents in organic synthesis [1, 2]. In addition to their synthetic applications, these compounds are fast gaining contemporary interest due to their indispensable applications in electronic industry [3], as organic conductors [4] and precursors for semiconducting materials [5], in biology [6] and in medical imaging.

It is curious to note that the chemistry of alkyl, aryl, and mixed alkyl aryl chalcogenides has developed rapidly for the last two decades and is of immense interest to organic chemists [7] and biochemists [8], whereas the chemistry of pyridyl derivatives virtually remained neglected, in spite of its greater utility [9]. Recently, the chemistry of pyridyl derivatives has attracted the attention of the scientific community due to their unique properties, which endear them to the new and exciting applications in organic synthesis. In recognition of its importance, renewed efforts have evolved for the convenient methodologies of their synthesis.

The presence of nitrogen in the aromatic ring brings remarkable changes and has attracted considerable attention of the practicing chemists as precursors in pharmacological compounds [10], for the preparation of liquid crystals [11], in the synthesis of polymers, and as ligands in transition metal complexes.

## 2. EXPERIMENTAL

All the manipulations were carried under a dry and deoxygenated nitrogen atmosphere to prevent the oxidation of oxygen-sensitive intermediates. Elemental sulphur, selenium, and tellurium (Sigma-Aldrich, Bangalore, India) were stored in a desiccator prior to use. Tetrahydrofuran (THF) was dried using sodium and benzophenone prior to use. Diisopropylamine (DIA) was distilled using CaH_2_ and was stored on molecular sieves. 2-halopyridines (Halo- = F, Cl, and Br), *n*-butyl lithium of analytical grade were purchased from Aldrich and used without further purification. ^1^H, ^13^C, and ^77^Se NMR spectra were recorded on a Jeol AL spectrometer operating at 300, 75.432, 57.203, and 94.790 MHz, respectively in CDCl_3_, using Me_4_Si as an internal standard for ^1^H and ^13^C NMR. Me_2_Se and Me_2_Te were used as an external reference for ^77^Se and ^125^Te NMR. IR spectra were obtained between KBr plates on a Perkin-Elmer model 1430. C, H, and N analyses were performed on a Perkin-Elmer 2400 CHN analyzer. Mass spectra were obtained on a VG-70S11-250J mass spectrometer. Separation and purifications of compounds were carried out using column chromatography performed on activated silica gel using hexane/ethyl acetate as eluant.

## 3. SYNTHESIS OF 2-HALO-3-PYRIDYL CHALCOGEN COMPOUNDS (X = F, CL, AND BR)

A 100 mL three-necked round bottom flask was charged with 20 mL dry THF and 3 mL (22 mmol) DIA under nitrogen and was cooled to −20°C. To this solution was added dropwise 17.6 mL (22 mmol, 1.25 M) *n*-butyl lithium with continuous stirring and the mixture was allowed to stand for 1 hour at 0°C. To this solution of lithium diisopropylamine (LDA) was added slowly a solution of 2-halopyridine (20 mmol) in THF (10 mL) at −50°, −40°, and −78°C in case of fluoro-, chloro-, and bromopyridine, respectively. Stirring was continued for additional one hour after which dry and activated elemental chalcogen (S, Se, and Te) (20 mmol) was added in small portions with continuous stirring. The temperature was raised slowly up to room temperature. It was found that sulphur and selenium takes 20–30 minutes to dissolve completely, while tellurium takes nearly one hour to dissolve, owing to surface oxidation of the metal. The product was hydrolyzed using 5 mL solution of aqueous ammonium chloride and nitrogen supply was discontinued. The reaction was then subjected to aerial oxidation for 10–12 hours in case of selenols and tellurols. Thiols, obtained from the hydrolysis of thiolate, gave a poor yield of disulfides upon aerial oxidation. Therefore, oxidation of thiols was performed using dimethyl sulfoxide (DMSO) at 80–90°C, which is a mild and successful oxidizing agent for the oxidation of thiols to disulfides. THF was removed on rota-evaporator; the resulting mixture was diluted with water and was extracted in dichloromethane (3 × 40 mL). Inorganic impurities were removed by repeatedly washing the organic layer with brine (3 × 30 mL) followed by distilled water. The organic extract was then dried over anhydrous sodium sulfate and concentrated on rota-evaporator. The product was purified on a silica column using 10% ethyl acetate-hexane as eluant.

## 4. RESULTS AND DISCUSSION

**Scheme 1:**
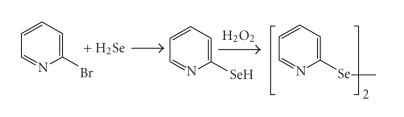


Mautner et al. [12] were the first to prepare *bis*(2-pyridyl) diselenide by reacting 2-bromo pyridine with toxic hydrogen selenide (see Scheme 1).

**Scheme 2:**
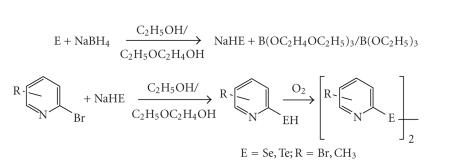


Toshimitsu et al. [13, 14] modified this synthesis circumventing the use of toxic hydrogen selenide by using sodium hydrogen selenide obtained by the reacting elemental selenium with sodium borohydride in 2-ethoxyethanol. Bhasin et al. [15] have optimized the use of this reagent for the synthesis of various substituted methyl- and bromopyridyl selenium and tellurium compounds (see Scheme 2).

**Scheme 3:**
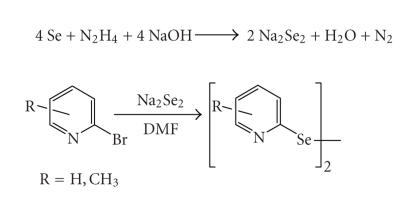


Syper and Mlochowski [16] developed a new methodology for the synthesis of *bis*(2-pyridyl) diselenide by reacting dilithium diselenide with 2-bromopyridine. Various methyl substituted 2-pyridyl diselenides/ditellurides were synthesized by Bhasin and Singh [17] using a mild and easily available reducing agent, hydrazine hydrate (see Scheme 3).

Engman and Cava [18] prepared *bis*(2-pyridyl) ditelluride through lithium bromine exchange of 2-bromopyridine using sterically hindered and highly reactive *t*-butyl lithium at −78°C in THF. The preparation of methyl substituted *bis*(2-pyridyl) diselenides and ditellurides were extended by Bhasin et al. [19] using metal-halogen exchange of methyl-substituted bromopyridines using *n*-butyl lithium.

Heteroatom-directed aromatic lithiation is a versatile route towards the synthesis of *π*-deficient heterocycles [20]. The presence of C−X bond in 2-halopyridines, apart from allowing easy and selective metalation at *ortho*-position, makes it potentially reactive towards nucleophiles, allowing the introduction of other functional groups. Metalation at *ortho*-position is facilitated owing to the *ortho*-directing ability of halogen substituent, particularly fluorine and chlorine. Such an intermediate is potentially reactive towards electrophilic selenium and tellurium metals. Therefore, design of new methods allowing metalation of 2-halopyridine for chalcogen incorporation with the retention of C−X bond could be of great synthetic value.

Among the various organolithium reagents, LDA has been known to bring about selective deprotonation as it is a nonnucleophilic base and does not lead to metal-halogen exchange reactions in halogenopyridines, which occur with *n*-butyl lithium.

**Scheme 4:**
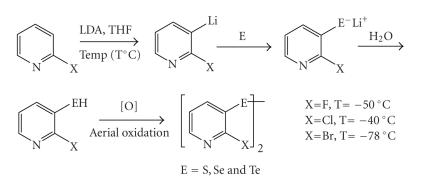
Protocol for regioselective synthesis of *bis*(2-halo-3-pyridyl) dichalcogenides.

In an effort to achieve the synthesis of target compounds, the deprotonation of 2-halopyridines (X= F, Cl, Br) was carried out under cryogenic conditions in THF using LDA as base. Reports from literature indicate that LDA can be used efficiently to induce exclusive lithiation at C-3 position [21] as a consequence of DoM effect (directed ortho-metalation). It was observed that the use of LDA prevented nucleophilic addition of base on C=N bond as well as metal-halogen exchange reactions. The intermediate, 3-lithio-2-haolpyridine, generated *in situ* was reacted with elemental chalcogen (S, Se, and Te) at low temperature. The insertion of chalcogen atom into C−Li bond took place readily resulting in the formation of 2-halo-3-pyridylchalcogenolate (Scheme 4). It was found that sulfur and selenium undergo smooth insertion into the C−Li bond while tellurium takes time to undergo insertion. This is possibly due to the metallic character and passive nature of this element. The resulting solution of 2-halo-3-pyridylchalcogenolate was subsequently subjected to hydrolysis. The oxidative coupling of resulting thiols, selenols, and tellurols affords the desired *bis*(2-halo-3-pyridyl) dichalcogenide in good yield.

**Scheme 5:**
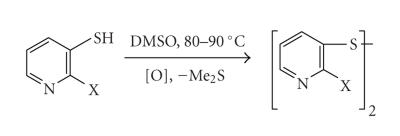
Oxidation of pyridine thiols to disulfides.

Simple aerial oxidation was sufficient to obtain diselenides and ditellurides, but thiols had to be oxidized using DMSO to get a quantitative yield of the desired disulfide (Scheme 5).

In order to ascertain the applicability of this protocol for the synthesis of various 2-halo-3-pyridyl chalcogenides, a series of reactions was set up. The results obtained revealed that the methodology was best applicable to chloro- and fluoro- derivatives. The yield was lowered to less than half in case of bromoderivatives. Insertion of tellurium in 2-bromo-3-lithiopyridine gave a poor result to the extent that even the recovery of a substantial amount of compound, sufficient for characterization, was not possible.

## 5. SPECTROSCOPIC STUDIES

The compounds prepared (Table 1) were characterized with the help of various spectroscopic techniques, *namely*, ^1^H (Table 2), ^13^C (Table 3), ^77^Se/^125^Te NMR (Table 4), IR, UV-Vis spectroscopy, mass spectrometry, and X-ray crystallographic techniques.

## 6. ^1^H NMR STUDIES

^1^H NMR spectra of hitherto unknown *bis*(2-halo-3-pyridyl) dichalcogenides were obtained in CDCl_3_ using TMS as internal reference. The NMR characterization of dichalcogenides along with the data has been given in Table 2. It was observed that the ^1^H NMR spectra for the dichalcogenides display three different sets of protons in aromatic region. In case of *bis*(2-chlolo-3-pyridyl) diselenide, H-6 proton appears most downfield and lies in 8.22–8.24 ppm while the signals corresponding to H-4 and H-5 appear at lower frequencies and fall in the regions 7.88–7.91 ppm and 7.15–7.19 ppm, respectively. The order of chemical shift values of pyridyl protons follows the order H-6 > H-4 > H-5. To substantiate these predictions further, [^1^H−^1^H] COSY studies were performed on the newly synthesized compounds.

## 7. [^1^H−^1^H] COSY (HOMCOR-2D) AND [^1^H−^13^C] COSY (HETCOR-2D) STUDIES

**Figure 1:**
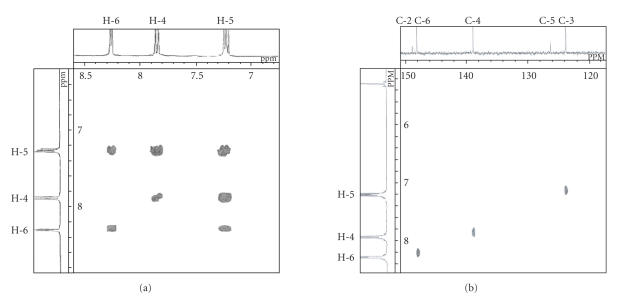
(a) [^1^H−^1^H] COSY (HOMCOR-2D) spectrum of *bis*(2-chloro-3-pyridyl) diselenide, (b) [^1^H−^13^C] COSY (HETCOR-2D) spectrum of *bis*(2-chloro-3-pyridyl) diselenide.

2D correlation spectroscopy helps in the assignment of protons and carbon signals in NMR spectrum, besides providing vital information about proton-proton and proton-carbon connectivities. The off-diagonal contours (cross-peaks) allow the identification of proton signals and help in interpreting ^13^C NMR spectrum. [^1^H−^1^H] COSY spectrum clearly shows a correlation of H-5 proton with H-4 and H-6 due to its *ortho*-position with respect to both. [^1^H−^13^C] COSY (HETCOR) correlates the peaks of a proton spectrum with the peaks of ^13^C spectrum (Figure 1).

^13^C peaks have unequivocally been sorted out with the help of off-diagonal cross-peaks corresponding to ^1^J_C−H_ coupling interactions. Accordingly, the assignments lie in the order of chemical shift as under C-2 > C-6 > C-4 > C-3 > C-5.

## 8. ^13^C NMR STUDIES

As evident from the ^13^C NMR studies of 2-halopyridines, the carbon-13 signals resonate downfield with the increasing electronegativity (F > Cl > Br) of halogen atom. Carbon atom directly bonded to the halogen experiences maximum deshielding due to −I effect of halogen. However, the inductive effect decreases from fluorine to bromine resonance effect (+R) increases; the carbon atom at *para* position with respect to halogen (C-5) shows the reverse trend in observed chemical shift values.

It appears that in the newly synthesized *bis*(2-halo-3-pyridyl) dichalcogenides, due to the opposing nature of inductive and resonance effect of chalcogen and halogen atoms, no such generalizations can be made. The interpretations of [^1^H−^1^H] and [^1^H−^13^C] COSY (Figure 1) studies and ^13^C NMR data reveal that in diselenides, C-2 carbon resonates most downfield relative to TMS.

## 9. ^77^Se NMR STUDIES

^77^Se NMR of *bis*(2-halo-3-pyridyl) diselenides (X = F, Cl, Br) were recorded in CDCl_3_ employing Me_2_Se as external reference (*δ*, 0 ppm). It is curious to note that with the increase in the electronegativity of halogen at C-2 position, an upfield shift is observed. It is also worthwhile to mention that all these compounds exhibit ^77^Se resonance at higher frequency relative to *bis*(2-pyridyl) diselenide. An answer may lie in the existing intermolecular short contacts operating in the molecule as evident from the solid-state structural studies.

## 10. ^125^Te NMR STUDIES

Chemical shifts are cited with respect to neat Me_2_Te (*δ* = 0 ppm) as external reference.

## 11. ^19^F NMR STUDIES

The proton-noise decoupled ^19^F NMR spectra of the fluorinated derivatives were recorded in deuterated chloroform, CDCl_3_, using trichloroflouromethane, CFCl_3_ (freon-11) as the external reference. The ^19^F signals for both compounds, *bis*(2-fluoro-3-pyridyl) diselenide and bis(2-fluoro-3-pyridyl) ditelluride, were observed as well-defined signals at −50.62 and −59.3 ppm.

## 12. MASS SPECTROMETRY

The isotopic richness of natural selenium and tellurium helps in the identification of selenium and tellurium containing fragments in the mass spectra of organoselenium and organotellurium compounds. A number of characteristic ions found in the mass spectra have been tabulated in Table 5.

## 13. IR STUDIES

This technique has been used for the general characterization of the newly prepared pyridyl selenium and tellurium compounds. IR spectra of these compounds were recorded in the range 4000–400 cm^−1^ in compressed transparent pellets made from powdered compounds and dry KBr. IR spectra of various compounds synthesized have been summarized in Table 6.

## 14. MOLECULAR GEOMETRY AND CRYSTAL STRUCTURE OF *BIS*(2-CHLORO-3-PYRIDYL) DISELENIDE

**Figure 2:**
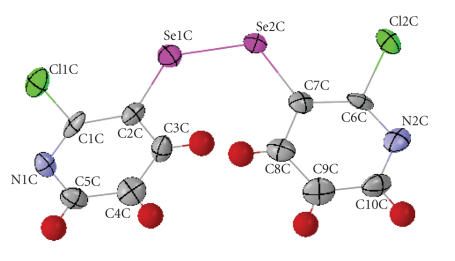
A perspective view of the structure of *bis*(2-chloro-3-pyridyl) diselenide.

To understand the structural details, single crystal X-ray diffraction analysis of *bis*(2-chloro-3-pyridyl) diselenide was carried out. A perspective view of the structure of this compound is shown in Figure 2. The selected bond parameters are listed in Tables 7 and 8. The molecule crystallizes in monoclinic, P2_1_/c space group:

(1) *a* = 11.390(2) Å, *b* = 27.851(5) Å,
*c* = 11.849(2) Å, *α* = 90°, *β* = 112.984(3)°, *γ* = 90°.

## 15. MOLECULAR STRUCTURE OF BIS(2-CHLORO-3-PYRIDYL) DITELLURIDE

Beautiful and bright orange colored, diamond-shaped single crystals of *bis*(2-chloro-3-pyridyl) ditelluride were obtained by a slow evaporation of dichloromethane and hexane (1 : 2). X-ray single crystal analysis of a selected specimen was done on Bruker Smart CCD diffractometer.

The compound crystallizes into monoclinic, C2/c space group:

(2) *a* = 11.6112(14) Å, *b* = 9.7812(12) Å, *c* = 12.0760(14) Å, *α* = 90°, *β* = 113.717(2)°, *γ* = 90°.

**Figure 3:**
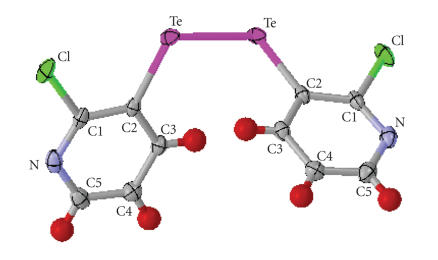
A perspective view of the structure of *bis*(2-chloro-3-pyridyl) ditelluride.

The ORTEP diagram of the compound is given in Figure 3 and important bond parameters are given in Tables 9 and 10.

## Figures and Tables

**Table 1:** Physical properties and analytical data of various 2-halo-3-pyridyl chalcogen compounds.

Compound	Physical state	Melting point (°C)	Yield (%)	C	H	N

*Bis*(2-chloro-3-pyridyl) disulfide	Colorless crystalline solid	160*	52	41.11 (41.52)	1.95 (2.07)	9.25 (9.68)
*Bis*(2-chloro-3-pyridyl) diselenide	Yellow diamond-shaped crystals	206-207	54	30.89 (31.16)	1.22 (1.55)	7.02 (7.27)
*Bis*(2-chloro-3-pyridyl) ditelluride	Orange-diamond shaped crystals	178–180	50	24.63 (24.74)	1.17 (1.23)	5.38 (5.77)
*Bis*(2-fluoro-3-pyridyl) diselenide	Pale yellow diamond-shaped crystals	62-63	65	33.82 (34.09)	1.54 (1.70)	7.49 (7.95)
*Bis*(2-fluoro-3-pyridyl) ditelluride	Red crystalline solid	55–59	58	26.13 (26.78)	1.09 (1.32)	6.01 (6.25)
*Bis*(2-bromo-3-pyridyl) diselenide	Orange crystalline powder	152–155	35	25.15 (25.31)	1.12 (1.26)	5.75 (5.90)

*Decomposes at 160°C.

**Table 2:** ^1^H NMR data of various 2-halo-3-pyridyl chalcogen compounds.

Entry	Compound	^1^H NMR (*δ*, ppm)		
		H-4	H-5	H-6

1	*Bis*(2-chloro-3-pyridyl) disulfide	7.83–7.86 (dd, 2H, 7.8, 1.8 Hz)	7.21–7.26 (dd, 2H, 7.8, 4.5 Hz)	8.24–8.26 (dd, 2H, 4.5, 1.8 Hz)
2	*Bis*(2-chloro-3-pyridyl) diselenide	7.88–7.91 (dd, 2H, 7.8, 1.8 Hz)	7.15–7.19 (dd, 2H, 4.8, 7.8 Hz)	8.22–8.24 (dd, 2H, 4.8, 1.8 Hz)
3	*Bis*(2-chloro-3-pyridyl) ditelluride	7.90–7.93 (dd, 2H, 4.5, 7.8 Hz)	6.95–6.99 (dd, 2H, 4.8, 7.5 Hz)	8.20–8.22 (dd, 2H, 7.8, 1.8 Hz)
4	*Bis*(2-fluoro-3-pyridyl) diselenide	7.03–7.08 (m, 2H)	7.91–8.03 (H-5, H-6) (m, 2H)	—
5	*Bis*(2-fluoro-3-pyridyl) ditelluride	7.00–7.06 (m, 2H)	8.03–8.15 (H-5, H-6) (m, 4H)	—
6	*Bis*(2-bromo-3-pyridyl) diselenide	7.79–7.82 (dd, 2H, 7.8, 1.8 Hz)	7.18–7.22 (m, 2H)	8.16–8.18 (dd, 2H, 4.8, 1.8 Hz)

**Table 3:** ^13^C NMR data of various 2-halo-3-pyridyl chalcogen compounds.

Entry	Compound	^13^C NMR (chemical shift)				

		C-2	C-3	C-4	C-5	C-6
1	*Bis*(2-chloro-3-pyridyl) disulfide	147.59	131.76	123.42	135.26	147.47
2	*Bis*(2-chloro-3-pyridyl) diselenide	148.93	126.48	124.01	139.00	148.11
3	*Bis*(2-chloro-3-pyridyl) ditelluride	152.74	106.44	124.54	146.10	149.18
		162.15	123.25	—	—	—
4	*Bis*(2-fluoro-3-pyridyl) diselenide	159.05	122.80	143.66	147.12	147.30
		(*d*, *J* = −234.05 Hz)	(*d*, *J* = 33.97 Hz)	—	—	—
5	*Bis*(2-fluoro-3-pyridyl) ditelluride	164.4, 161.3	88.1, 87.5	123.2	148.0	150.6
		(*d*, *J* = −234.05 Hz)	(*d*, *J* = 45.8 Hz)	—	—	—
6	*Bis*(2-bromo-3-pyridyl) diselenide	148.50	127.53	124.39	138.26	148.28

**Table 4:** ^77^Se/^125^Te NMR data of various 2-halo-3-pyridyl chalcogen compounds.

Entry	Compound	^77^Se/^125^Te
		(*δ*, ppm)

1	*Bis*(2-chloro-3-pyridyl) diselenide	387.1
2	*Bis*(2-fluoro-3-pyridyl) diselenide	370.3
3	*Bis*(2-bromo-3-pyridyl) diselenide	407.1
4	*Bis*(2-chloro-3-pyridyl) ditelluride	7.5
5	*Bis*(2-fluoro-3-pyridyl) ditelluride	419.8

**Table 5:** Mass spectral data of *bis*(2-chloro-3-pyridyl) diselenide/ditelluride.

Entry	Compound	Stands for Mass/electron	Relative intensity	Assignment

1	*Bis*(2-chloro-3-pyridyl) diselenide	385	47.1	[M]^+^
		304	4.6	[(ClPy)_2_^80^Se]^+^
		192	100	[ClPy^80^Se]^+^
		157	10.6	[Py^80^Se]^+^
		77	3.7	[PyH]^+^

2	*Bis*(2-chloro-3-pyridyl) ditelluride	485	23.4	[M]^+^
		355	11.6	[(ClPy)_2_^130^Te]^+^
		242	42.5	[ClPy^130^Te]^+^
		207	3.5	[Py^130^Te]^+^
		77	100	[PyH]^+^

**Table 6:** Infrared spectral data of various 2-halo-3-pyridyl chalcogen compounds.

Entry	Compound	KBr (cm^−1^)

1	*Bis*(2-chloro-3-pyridyl) disulfide	3097.6, 3045.5, 2924.8, 1736.9,
		1555.2, 1431.6, 1260.0, 1212.1,
		1143.2, 1059.4, 796.0, 750.8,
		723.2, 655.9, 517.9, 438.1, 474.0

2	*Bis*(2-chloro-3-pyridyl) diselenide	3031.8, 1957.3, 1735.1, 1596.8,
		1553.8, 1428.3, 1255.9, 1208.3,
		1124.1,1055.7, 793.8, 743.3,
		721.3, 642.1, 510.2, 441.4

3	*Bis*(2-chloro-3-pyridyl) ditelluride	3018.7, 1608.0, 1547.5, 1424.4,
		1368.4, 1250.5, 1203.0, 1007.3,
		791.8, 725.1, 720.9, 634.8, 502.4

4	*Bis*(2-fluoro-3-pyridyl) diselenide	3036.7, 1946.8, 1720.5, 1576.7,
		1555.0, 1411.9, 1290.1, 1226.6,
		1126.8, 1063.4, 1025.8, 834.1,
		792.0, 731.8, 653.9, 564.1

5	*Bis*(2-fluoro-3-pyridyl) ditelluride	2924.8, 2853.3, 1574.3, 1555.7,
		1415.6, 1245.8, 1066.0, 1018.4,
		788.1, 646.3, 566.2

6	*Bis*(2-bromo-3-pyridyl) diselenide	3018.6, 2925.9, 2853.9, 1736.4,
		1552.4, 1599.0, 1467.9, 1426.3,
		1377.1, 1214.8, 1052.3, 762.0,
		668.8, 627.5, 497.2

**Table 7:** Important bond lengths [Å] and angles [ ° ] for *bis*(2-chloro-3-pyridyl) diselenide.

Se(1A)−C(2A)	1.926(8)	Se(1C)−Se(2C)	2.3003(13)
Se(1A)−Se(2A)	2.2973(13)	Se(2C)−C(7C)	1.898(9)
Se(2A)−C(7A)	1.912(9)	Cl(1A)−C(1A)	1.734(8)
C(2A)−Se(1A)−Se(2A)	102.0(2)	C(5A)−N(1A)−C(1A)	116.2(8)
C(7A)−Se(2A)−Se(1A)	102.3(2)	C(1B)−N(1B)−C(5B)	116.7(8)
C(2B)−Se(1B)−Se(2B)	101.8(2)	C(6B)−N(2B)−C(10B)	115.8(9)

**Table 8:** Important torsion angles [ ° ] of *bis*(2-chloro-3-pyridyl) diselenide.

C(2A)−Se(1A)−Se(2A)−C(7A)	83.1(3)	Se(1B)−C(2B)−C(3B)−C(4B)	176.6(6)
C(2B)−Se(1B)−Se(2B)−C(7B)	−82.7(3)	N(2B)−C(6B)−C(7B)−Se(2B)	−178.1(7)
C(2C)−Se(1C)−Se(2C)−C(7C)	−84.6(4)	Cl(2B)−C(6B)−C(7B)−Se(2B)	1.4(9)
Se(1A)−C(2A)−C(3A)−C(4A)	−177.3(6)	Se(1C)−C(2C)−C(3C)−C(4C)	176.7(7)
N(1A)−C(1A)−C(2A)−Se(1A)	178.0(6)	N(1C)−C(1C)−C(2C)−Se(1C)	−176.8(7)

**Table 9:** Important bond lengths [Å] and angles [ ° ] for *bis*(2-chloro-3-pyridyl) ditelluride.

Te−C(2)	2.124(2)	N−C(5)	1.339(4)
Te−Te#1	2.6771(4)	C(1)−C(2)	1.396(3)
Cl−C(1)	1.754(3)	C(2)−C(3)	1.387(3)
N−C(1)	1.313(3)	C(3)−C(4)	1.385(3)
C(2)−Te−Te#1	99.33(6)	N−C(1)−Cl	115.62(17)
C(1)−N−C(5)	116.3(2)	C(2)−C(1)−Cl	118.0(2)
N−C(1)−C(2)	126.4(2)	C(3)−C(2)−Te	124.50(17)

**Table 10:** Important torsion angles [ ° ] of *bis*(2-chloro-3-pyridyl) ditelluride.

C(5)−N−C(1)−C(2)	−0.3(4)	Te#1−Te−C(2)−C(1)	−168.01(17)
C(5)−N−C(1)−Cl	180.00(18)	Te#1−Te−C(2)−C(3)	16.7(2)
N−C(1)−C(2)−Te	−174.83(19)	Te−C(2)−C(3)−C(4)	174.54(17)
Cl−C(1)−C(2)−Te	4.8(3)	—	—
